# Mechanical ventilation weaning issues can be counted on the fingers of just one hand: part 1

**DOI:** 10.1186/s13089-020-00161-y

**Published:** 2020-03-13

**Authors:** Luigi Vetrugno, Giovanni Maria Guadagnin, Alessandro Brussa, Daniele Orso, Eugenio Garofalo, Andrea Bruni, Federico Longhini, Tiziana Bove

**Affiliations:** 1 Anesthesiology and Intensive Care Clinic, Department of Medicine, University of Udine, Via Colugna 50, 33100 Udine, Italy; 2 Intensive Care Unit, Department of Medical and Surgical Sciences, University Hospital Mater Domini, Magna Graecia University, Catanzaro, Italy

**Keywords:** Weaning, Mechanical ventilation, Ultrasound, Echocardiography, Diaphragm, Diaphragm dysfunction

## Abstract

Although mechanical ventilation may be a patient’s vital ally during acute illness, it can quickly transform into an enemy during chronic conditions. The weaning process is the fundamental phase that enables the resumption of physiological respiratory function; however, it is also associated with a number of life-threatening complications, and a large percentage of critically ill patients never achieve airway device removal or require the resumption of mechanical ventilation just a few days post-weaning. Indeed, the weaning process is, at present, more of an art than a science. As such, there is urgent need for novel contributions from the scientific literature to abate the growing rates of morbidity and mortality associated with weaning failure. The physician attempting to wean a patient must integrate clinical parameters and common-sense criteria. Numerous studies have striven to identify single predictive factors of weaning failure and sought to standardize the weaning process, but the results are characterized by remarkable heterogeneity. Despite the lack of benchmarks, it is clear that the analysis of respiratory function must include a detailed overview of the five situations described below rather than a single aspect. The purpose of this two-part review is to provide a comprehensive description of these situations to clarify the “arena” physicians are entering when weaning critically ill patients from mechanical ventilation.

## Introduction

Up to 25% of critically ill patients experience difficulties in weaning from invasive mechanical ventilation (IMV), burdening the intensive care unit (ICU) with long lengths-of-stay, prolonged mechanical ventilation (IMV), and increased morbidity and mortality [1]. Weaning failure is commonly described as a failed spontaneous breathing trial (SBT) or the reinstitution of mechanical ventilation within 2 to 7 days after extubation [2]. Failed extubation complicates up to 30% of successful spontaneous breathing trials [3] and correlates with higher morbidity and mortality rates [4, 5].

Determining the optimal time to extubate a patient continues to be a challenge. Prolonged mechanical ventilation is associated with a series of adverse effects (e.g., tracheal lesions, ventilator-associated pneumonia, ventilator-induced lung injury, and ventilator-induced diaphragm injury) [6, 7], whereas premature extubation exposes the patient to cardiovascular stress due to spontaneous breathing, often requiring the reinstitution of ventilatory support [8]. Although international guidelines recommend the implementation of spontaneous breathing trials [9], used alone this practice is ineffective in predicting weaning failure and reintubation [10–13]. A number of studies have investigated and proposed different parameters as outcome predictors of weaning from mechanical ventilation; however, a clear understanding of the ideal time to proceed with extubation for a successful outcome continues to elude us [14, 15]. Cardiac failure, diaphragmatic dysfunction and acute respiratory illness (lung parenchyma disease, or upper or lower airway obstructions), neurological status, and intra-abdominal issues are among the known leading causes of failed weaning [3, 16].

Significant advancements have been achieved in ultrasound technology over the last 20 years, most notably in bringing these techniques to the bedside, with all the advantages that this conveys. In fact, our capacity to study the individual organs ultrasonographically has become so finely tuned and specialized that physicians risk overlooking a more integrated examination of the patient. A comprehensive approach to invasive mechanical ventilation should include an extensive ultrasonographic assessment of cardiac function, diaphragmatic activity, and lung parenchyma. In particular, much light has been shone on the use of ultrasonographic assessment of diaphragmatic dysfunction as a predictor of weaning failure. That said, it is fundamental that the diaphragm does not receive excessive attention at the expense of other predictive factors. A simultaneous and comprehensive ultrasound examination of the cardio-pulmonary-diaphragmatic district may confer valuable predictive information regarding successful weaning from mechanical ventilation, although literature in this regard is still limited [17]. It is also important to bear in mind that other factors able to negatively influence the weaning outcome exist outside the cardio-pulmonary-diaphragmatic area (i.e., the neurological status and intra-abdominal hypertension) that cannot be evaluated with ultrasound [18, 19].

Mechanical ventilation can negatively affect the actors involved in respiratory function; for example, ventilator-induced lung injury, impaired cardiac performance, and ventilator-induced diaphragmatic dysfunction are all possible consequences. Although the concept of ‘lung protection’ during mechanical ventilation is well established, a more precise definition of a ‘protective modality’ to help preserve all the elements involved in the respiratory function is also desirable.

The purpose of this two-part review is to illustrate the main causes of unsuccessful weaning and recent advancements on the application of bedside ultrasound evaluation in these topics.

## The weaning path

**Fig. 1:**
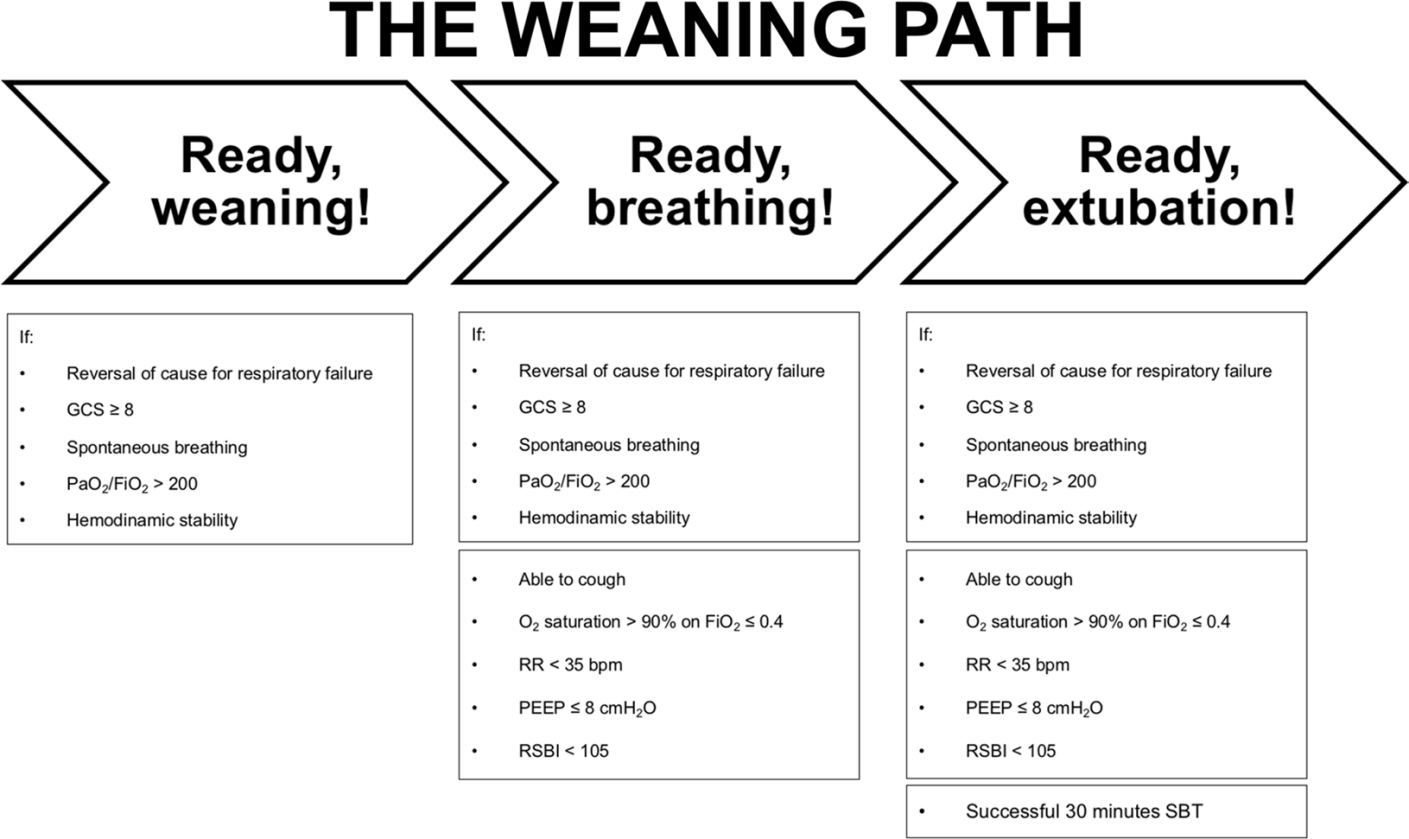
The weaning path. *GCS* Glasgow Coma Scale, *RR* respiratory rate, *PEEP* positive end-expiratory pressure, *RSBI* rapid shallow breathing index, *SBT* spontaneous breathing trial

Weaning from mechanical ventilation starts with the institution of mechanical ventilation; from the outset, the physician assesses the patient on a daily basis to determine when they might be ready to wean. Invasive mechanical ventilation is a risky journey for a critically ill patient admitted to the intensive care unit because of acute illness, IMV itself, or a failed weaning attempt. Weaning is the process in which the breathing effort is progressively returned from the machine to the patient. This process consists of 3 key steps, Fig. 1: first, when the patient’s clinical conditions permit it, ventilation supports are progressively decreased (*Ready, weaning*!); second, a spontaneous breathing trial (SBT) assesses the patient’s capability to breathe autonomously (*Ready, breathing*!); and third, the patient is liberated from mechanical ventilation (*Ready, extubation!*) [20, 21]. Three main SBT strategies are described in the literature: the T-piece trial; the continuous positive airway pressure (CPAP) trial—in which the CPAP applied is equal to the previously applied positive end-expiratory pressure (PEEP); and positive support ventilation, which uses a low level of pressure support (5–8 cmH_2_O).

No standardized SBT method has been identified to date in terms of type or duration (30–120 min) that can ensure a higher rate of successful weaning. However, strong evidence does exist supporting the employment of a short-term (30 min) breathing trail and low-level pressure support (8 cmH_2_O) [22]. If the SBT is accompanied by patient discomfort (i.e., anxiety or fatigue) and/or cardio-respiratory distress (i.e., RR ≥ 35 bpm, SpO_2_ < 90%, HR variability > 20%, hemodynamic instability) [23], then it is considered to have failed. In the absence of such responses, the SBT is deemed successful and the patient can be considered for airway removal (*Ready, extubing!*). Although heterogeneity exists in the definition of successful weaning, it is generally taken as the patient’s capacity to maintain spontaneous breathing for at least 48 h following extubation [3]. As mentioned above, despite international guidelines recommending the implementation of spontaneous breathing trials [3, 9], used alone SBTs are ineffective in predicting weaning failure and reintubation [10–13]. One possible solution to this may be the implementation of integrated cardio-pulmonary-diaphragmatic assessments for the detection of predictive indicators of weaning failure.

## The (he)art of weaning from mechanical ventilation

In the case of shock patients admitted to intensive care, most are resuscitated with the use of mechanical ventilation and intravenous fluid for respiratory and circulatory support. Regarding fluid management procedures in shock patients, recent literature has schematized its practice into four different phases, adopting the acronym ROSE: resuscitation (R), optimization (O), stabilization (S), and evacuation (E) [24]. Resuscitation, in some cases, could be too aggressive with the risk of fluid-overload, whereas evacuation could be too fast with the risk of hypovolemia and low cardiac output state (see below). In the first case, previous over-hydration with a positive fluid balance has been associated with death and an increased risk of failed weaning from mechanical ventilation in surviving patients [25, 26]. This could be explained by the fact that shifting the patient from mechanical ventilation (positive airway pressure) to a T-piece trial (negative airway pressure) can increase cardiac preload and unmask an underlying cardiac problem. Lemaire and colleagues were the first to demonstrate this vicious circle [8]. Potential cardiac complications can be monitored in 3 main ways: one is direct but invasive—measurement of the cardiac filing pressure by pulmonary arterial catheter (PAC); the remaining two are indirect and un-invasive—one entails the use of heart or lung ultrasound, and the other the rapid measurement of natriuretic peptide for the diagnosis of acute heart failure [27, 28].

**Fig. 2:**
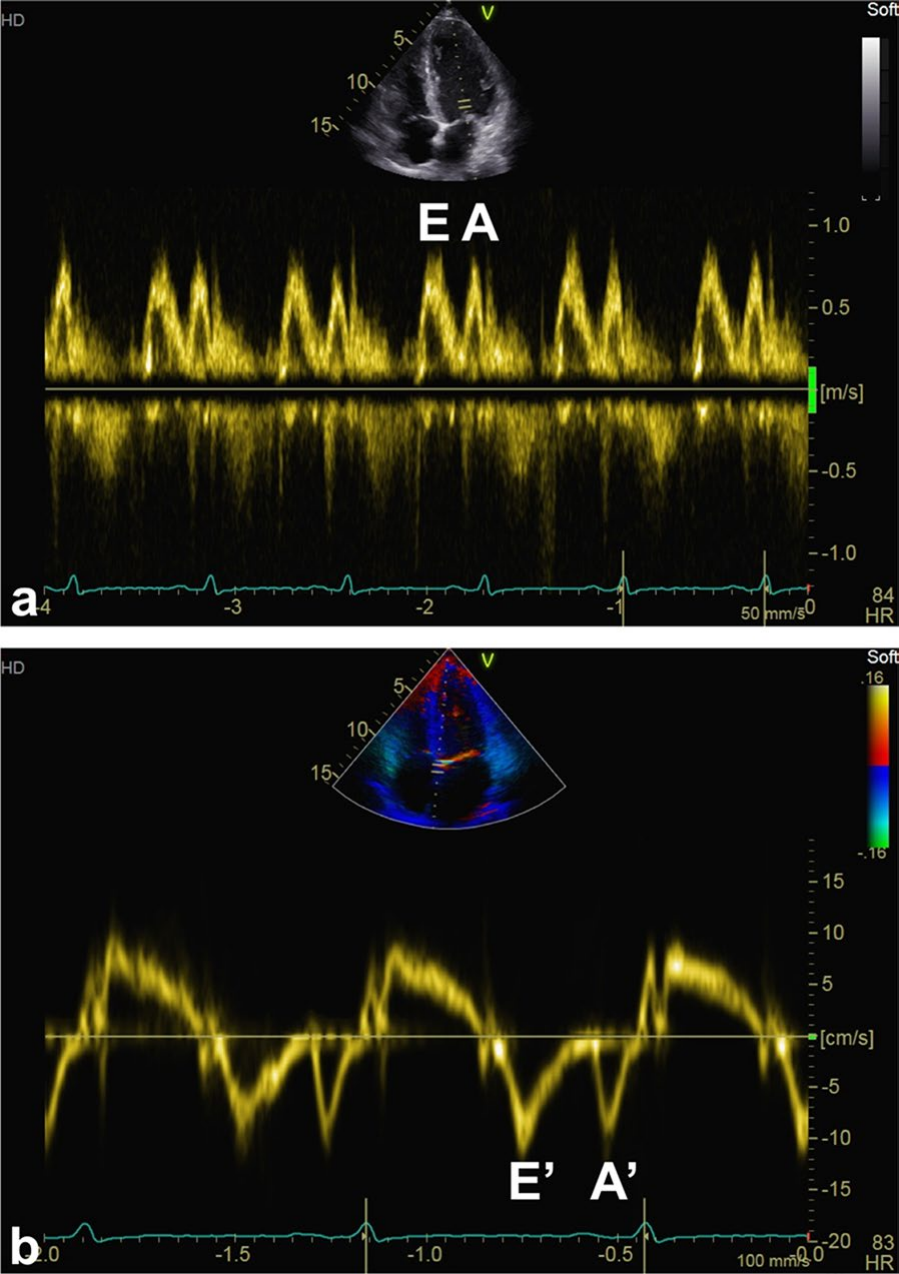
Pulse wave Doppler and tissue Doppler. **a** Trans-mitralic Doppler; an early wave (E wave) is displayed on the monitor that represents the first phase of the diastole as a passive phenomenon, limited only by the stiffness of the left ventricle or by interventricular interdependence—right ventricular push over the left ventricle via the septum. The E wave in patient with sinus rhythm is followed by atrial contraction (A wave). **b** Septal tissue Doppler; tissue Doppler directly measures myocardial velocities by means of the Eʹ wave in a load-independent way. The combination of the trans-mitral Doppler E wave, the tissue Doppler Eʹ wave, and the *E*/*E*ʹ ratio at the end of an SBT also seems to be capable of predicting weaning failure from diastolic dysfunction

Monitoring for cardiac complications is necessary because the sensitivity and specificity of clinical signs of acute heart failure in intensive care patients are poor [29]. PAC is reserved for specific severe cases. It is used for the diagnosis and treatment of pulmonary arterial hypertension and to guide fluid and catecholamine management; however, its routine use has been abandoned globally due to the lack of any outcome benefits [30, 31]. Ultrasound, on the other hand, has become an invaluable tool in the ICU thanks to its widespread availability at the patient’s bedside and the absence of any known side effects [28]. Moreover, the role of echocardiography in the detection of cardiac origins of weaning failure has grown significantly over the last 15 years. In particular, we now know that impaired systolic function (i.e., a small left ventricular ejection fraction [LVEF]) is less frequently associated with weaning failure in patients who failed the SBT (33% of cases) than left ventricular diastolic dysfunction (67% of cases) [28, 32, 33]. To investigate this latter condition, trans-mitral flow is interrogated using a pulse wave Doppler placed over the mitral valve in an apical four-chamber view. An early wave (E wave) is displayed on the monitor, Fig. 2a, that represents the first phase of the diastole as a passive phenomenon, limited only by the stiffness of the left ventricle or by interventricular interdependence—right ventricular push over the left ventricle via the septum. A stiff ventricle generates an elevated filling pressure, leading to pulmonary edema. In addition to trans-mitral Doppler, an E wave inpatient with sinus rhythm is followed by atrial contraction (A wave, Fig. 2a). The left ventricular preload is the sum of the E and A waves at a price of low or average left ventricular end-diastolic pressure (LVEDP) less than 12–14 mmHg. However, to estimate the LVEDP non-invasively, the E/A ratio has shown to be preload dependent and insufficient [34]. On the other hand, tissue Doppler directly measures myocardial velocities by means of the Eʹ wave in a load-independent way, Fig. 2b. The combination of the trans-mitral Doppler E wave, the tissue Doppler Eʹ wave, and the *E*/*E*ʹ ratio at the end of an SBT also seems to be capable of predicting weaning failure from diastolic dysfunction in patients with arrhythmia, immediately after and 10 min after extubation, with an area under the curve (AUC) of 0.75 and 0.86, respectively [33]. However, two main limitations of these methods need to be recognized. First, although an *E*/*E*ʹ value < 8 can accurately identify patients with a normal LVEDP, and an *E*/*E*ʹ value > 15 can identify those with an elevated LVEDP, a gray zone exists between the values of 8 and 15 [33–35]; second, not all intensive care clinicians possess advanced echocardiography skills; and third, LVEDP may also be elevated in cases of increased intra-abdominal pressure (see below).

In a very recent study, lung ultrasound evaluating the delta-B-lines before and after SBT in the four anterior chest wall regions was shown to provide excellent correlation with successful weaning [36]. However, the shift from mechanical to spontaneous ventilation may alone justify the appearance of such artifacts. Furthermore, distinguishing between cardiogenic and inflammatory causes of delta-B-lines may be a challenge.

Lastly, natriuretic peptides, and in particular brain natriuretic peptide (BNP), have been studied for their potential to predict weaning failure. In their study, Mekontso-Dessap et al. reported the outcome of two weaning strategies, one of which was driven by BNP level, whereas the other followed no explicit protocol [37]. They found that the BNP-guided fluid strategy was associated with increased use of diuretics, more negative fluid balance leading to a shorter mechanical ventilation time, and more ventilator-free days, but no change in ICU length-of-stay, morbidity, or mortality. The authors suggest that the changes in BNP that occur in the ICU are relatively slow and may be influenced by renal insufficiency or by the use of extracorporeal renal replacement therapy.

As previously mentioned, one-third of weaning failures with a cardiac origin depend on systolic dysfunction. In the case of cardiogenic shock, the diaphragmatic blood flow decreases and diaphragm muscular function can become compromised. In normal conditions, the diaphragmatic blood flow significantly increases with a high level of muscle contraction [38]. Indeed, the diaphragmatic blood flow is strictly dependent on cardiac output in both individuals at rest and with increased muscular activity [39]. Thus, since the diaphragm requires a high blood flow—in fact it is the skeletal muscle receiving the largest volume of blood per gram of tissue—diaphragm function may be impaired by a reduction in blood flow [40].

**Fig. 3:**
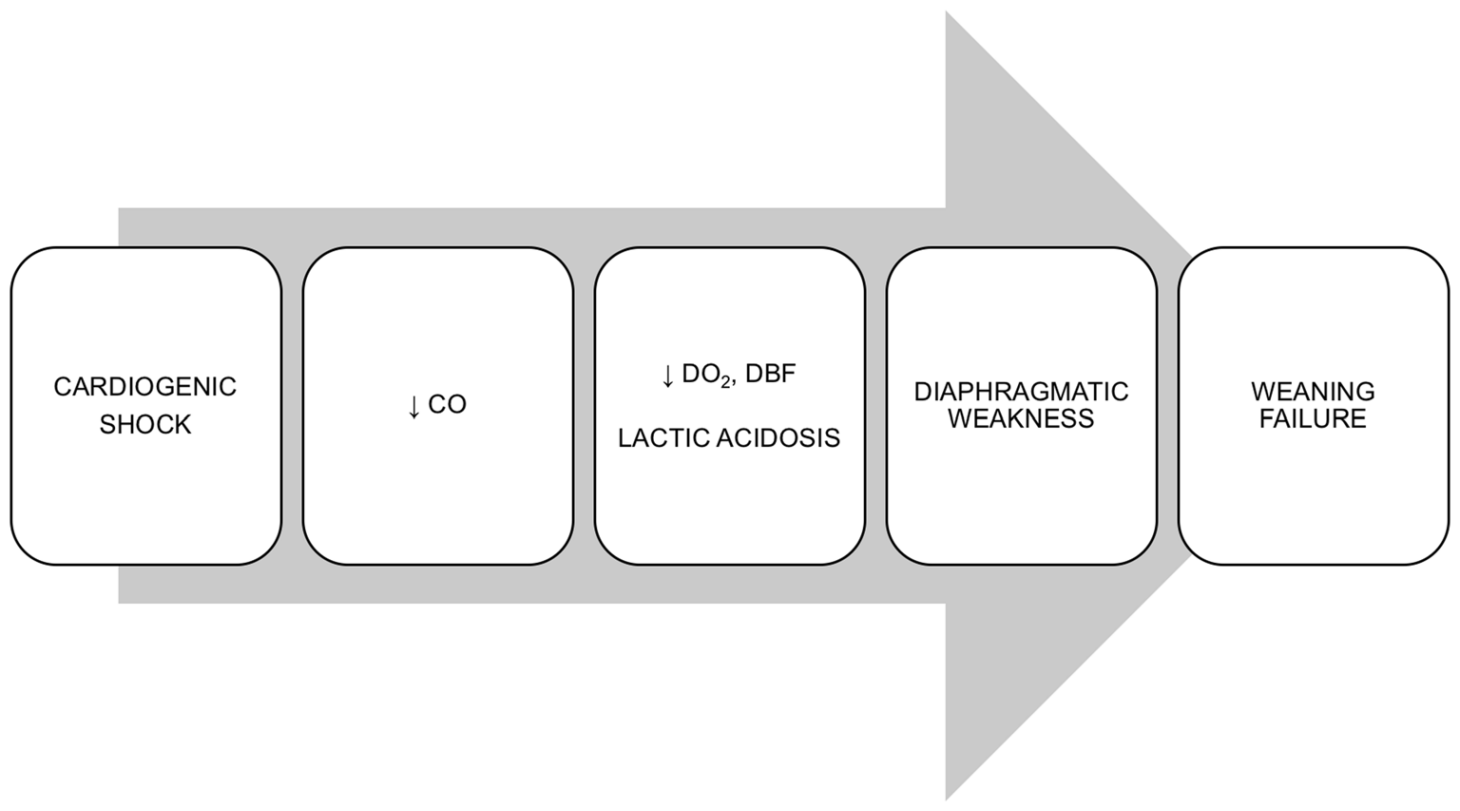
Cardiogenic shock impairing diaphragm activity. *CO* cardiac output, *DO*_*2*_ oxygen delivery, *DBF* diaphragm blood flow

In patients with cardiogenic shock, respiratory muscles, which are the only muscles working during low cardiac output, shift to anaerobic metabolism resulting in lactate production even though they may be receiving a large fraction of the cardiac output [41]. As a consequence, skeletal muscles and vital organs receive less blood flow, contributing to an increased production and impaired removal of lactate [41]. The onset of acidemia and hypoxemia then leads to an increase in the work of breathing due to induced compensatory hyperventilation. Of note, whenever the muscle oxygen and nutrient demands exceed the supply, the diaphragm’s ability to generate and sustain force decreases and muscle failure ensues [42]. During cardiogenic shock, the imbalance between demand and supply is facilitated by the reduced cardiac output and diaphragmatic blood flow (see Fig. 3).

In 1982, Aubier and colleagues investigated the influence of respiratory muscle activity on lactic acidosis and survival time in a cardiogenic shock canine model [41]. As opposed to mechanically ventilated dogs, the spontaneously breathing ones showed higher lactate levels and lower glycogen concentration due to the increased minute ventilation and respiratory workload occurring in the first hour after the onset of cardiogenic shock; afterward, minute ventilation rapidly decreased, due to diaphragmatic fatigue, till the death of the animal [41]. Furthermore, the use of neuromuscular blocking agents in mechanically ventilated dogs may have reduced lactate production due to the absence of diaphragmatic activity [41]. Impairment of the diaphragmatic contraction process during cardiogenic shock may be the underlying mechanism responsible for ventilatory failure (see below) [43]. No studies have so far evaluated the ultrasonographic modifications of the diaphragm during the course of or after cardiogenic shock, or their impact on short- and long-term outcomes.

## Diaphragm ultrasound (DUS)

The diaphragm, being the primary respiratory muscle, performs the majority of the work entailed in breathing. Diaphragm dysfunction, defined as the inability of the diaphragm to generate reasonable levels of maximal force, is an under-recognized pathological condition in critically ill patients and can render weaning from mechanical ventilation extremely difficult, resulting in protracted mechanical ventilation and prolonged intensive care unit length-of-stay [44]. The prevalence of diaphragm dysfunction in critically ill patients admitted to the intensive care unit and requiring IMV is over 60%, reaching 80% in patients requiring prolonged IMV and experiencing difficult weaning [45].

Considerable research attention has been focused on diagnosing diaphragm dysfunction over recent years, and there is strong evidence to sustain an association/correlation between diaphragm dysfunction and weaning failure [45–47]. The gold standard for evaluating diaphragm force is the measurement of trans-diaphragmatic pressure (Pdi) [48]. This test entails the insertion of a dedicated catheter with two balloons that measure the esophageal and gastric pressures, respectively. Diaphragm dysfunction occurs when Pdi < 11 cmH_2_O [48].

Another adjunctive test to assess diaphragm activity is the electrical activity of the diaphragm (EAdi) test. EAdi is performed using a dedicated esophageal catheter that has four couples of electrodes at its distal end. Besides monitoring the diaphragm, EAdi is also used to drive the ventilator with a proportional mode of ventilation, the so-called neutrally adjusted ventilator assist (NAVA). Thus, EAdi can be used to monitor the diaphragm at the bedside, whereas NAVA can exert a rehabilitative role in the case of diaphragm dysfunction.

Bedside ultrasound assessment of the diaphragm in critically ill patients can also be used to detect diaphragm dysfunction (diaphragm activity is classified as: usual, reduced, or loss of function) [49]. Although it may help the clinician make therapeutic decisions, a possible role in ventilation and weaning from mechanical ventilation has yet to be fully determined [49–52].

**Fig. 4:**
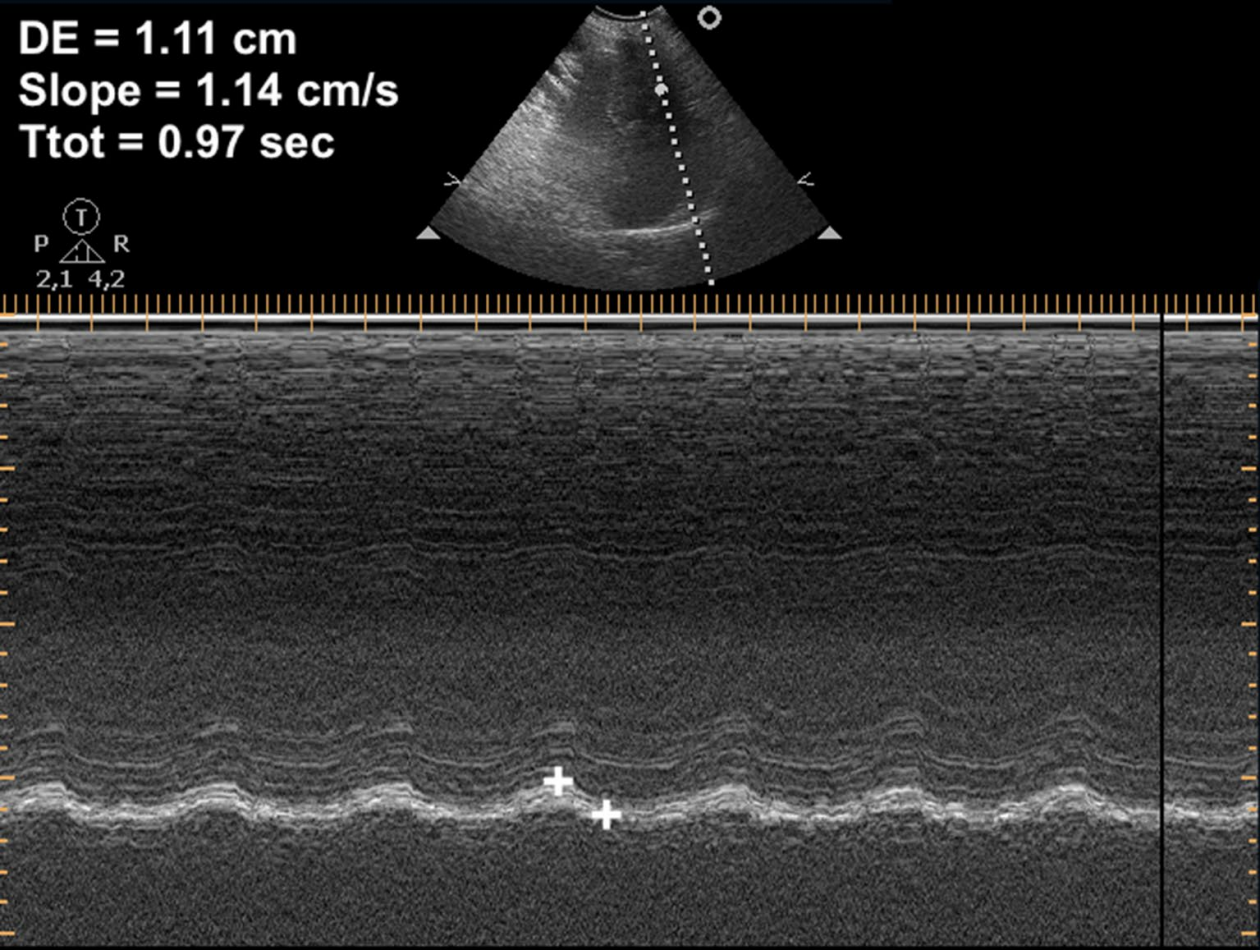
Diaphragm excursion. *DE* diaphragmatic excursion, *Slope* diaphragmatic contraction speed, *Ttot* inspiration + expiration time
- Diaphragmatic thickening fraction (DTF, %)—defined as the difference between the end-inspiration thickness (Te-Insp) and end-expiration thickness (Te-exp) divided by the end-expiration thickness (Fig. 5).

Two diaphragm sonographic predictors of successful weaning have been proposed [45, 49–56]:

- Diaphragmatic excursion (DE, cm)—defined as the maximum displacement of the hemidiaphragm during breathing (Fig. 4);

**Fig. 5:**
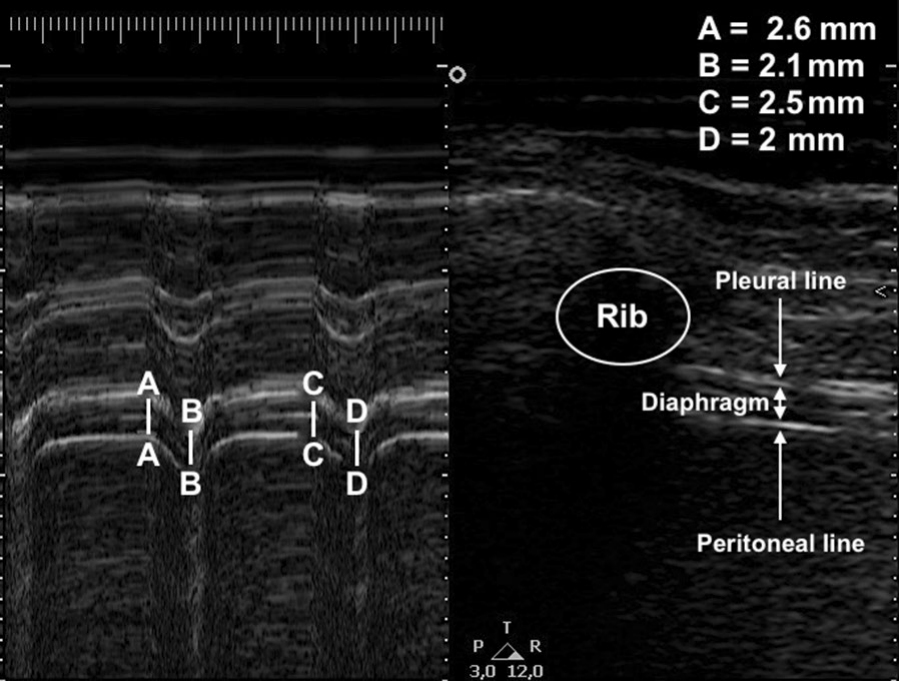
Diaphragm thickening fraction assessment. **a**, **c** End of inspiration thickness; **b**, **d** End of expiration thickness

Some studies have hypothesized that diaphragmatic excursion or liver/spleen movements could predict extubation failure. DE reflects the sum of the patient’s inspiratory activity and mechanical ventilatory support, so it only has real value when assessed during a spontaneous breathing trial. Diaphragm displacement is associated with lung volume during the inspiratory phase [57], but it does not correlate with and does not quantify the patient’s inspiratory effort [58]; furthermore, its assessment is influenced by numerous factors (see Table 1) [45]. It should also be noted that diaphragmatic excursion can be influenced by PEEP and levels of ventilator support during mechanical ventilation [55]: by increasing lung expiratory volumes, higher levels of PEEP lower the diaphragmatic domes, resulting in a decreased diaphragmatic excursion; during assisted ventilation, diaphragmatic excursion results from ventilator support plus the patient’s inspiratory efforts [56]. Therefore, only diaphragm thickening can provide a reliable indicator of diaphragm function during assisted mechanical ventilation [57].

**Table 1:** Most common bias to consider before an ultrasound assessment of diaphragmatic function

	Affected parameters	
	DE	DTF
Poor echographic transthoracic window		
Obesity	Ø	Ø
Splenectomy (left hemidiaphragm)	Ø	Ø
Altered starting position of hemidiaphragm		
Downward: COPD, asthma, PLEFF	Ø	
Upward: abdominal hypertension, ascites, supine position	Ø	
Iatrogenic impaired function		
Drugs (sedative, myorelaxants, etc.)	Ø	Ø
Mechanical ventilation		
Inspiratory positive pressure	Ø	
PEEP	Ø	
Operator-dependent factors		
*a* angle other than 90°	Ø	
ZOA identification^a^		Ø

*DE* diaphragmatic excursion, *DTF* diaphragm thickening fraction, *COPD* chronic obstructive pulmonary disease, *PLEFF* pleural effusion, *PEEP* positive end-expiratory pressure, *a angle* angle between M-Mode and hemidiaphragm examined, *ZOA* zone of apposition

^a^The further away from the ribs, the smaller the DTF will be

DTF, also known as the “ejection fraction” of the diaphragm, reflects active diaphragm contraction and inspiratory effort [59, 60]. It is associated with ICU length-of-stay, the duration of mechanical ventilation, and mortality [59, 60]. Whereas diaphragmatic excursion is not able to evaluate the degree of contractile activity of the diaphragm, DTF, on the other hand, has this capacity and can be used both in IMV (assisted or supported) and during an SBT [45].

A number of studies have investigated these two parameters, either individually or in combination, with the aim of identifying cut-off values as predictors of successful weaning (see Table 2) [43, 53, 61–73]. However, the results have been heterogeneous in terms of outcome results and cut-off values due to differences in study protocol design and outcome designation.

**Table 2:** Recent literature on predictive value of DUS

Variables	Study	Major findings
Diaphragm excursion (DE)	Jiang et al. [61]	Mean values of liver and spleen displacement during SBTs were higher in the successfully weaned group. Sensitivity and specificity for predicting successful extubation were 84.4% and 82.6%, respectively
	Spadaro et al. [62]	By replacing tidal volume (VTe) with DE in the formula for the Rapid Shallow Breathing Index (RSBI = RR/VTe), the authors created a new index called the diaphragmatic-RSBI (D-RSBI) The use of diaphragmatic excursion makes this index more accurate than the traditional rapid shallow breathing index in predicting outcome from weaning
	Kim et al. [43]	The authors describe an association between diaphragmatic dysfunction (DE < 10 mm obtained by M-Mode ultrasonography), a prolonged weaning period, and early or delayed weaning failures
	Luo et al. [63]	The authors find that a DE cut-off value < 12.6 mm was the most reliable predictor of reintubation within 1 week (sensitivity 80%, negative specificity 68.4%)
	Garrido-Aguirre et al. [64]	The authors propose a new weaning index (ULDIMex) that takes into consideration inspiration time (*a*), expiration time (*b*), and DE (*c*). With the conceived formula (*a* + *b*)*c*/2, the authors identified a cut-off slope value of 4.06 cm/s for ULDIMex in successfully weaned patients, with a negative predictive value of 96.5%
	Palkar et al. [65]	Greater diaphragm excursion, a longer inspiratory time, and a smaller decrease in the diaphragmatic excursion-time (E-T) index during SBT may help to predict successful extubation
	Carrie et al. [66]	According to the authors, DE was unable by itself to predict weaning failure with low sensitivity and specificity 59% and 71%, respectively
Diaphragm thickening fraction (DTF)	Jung et al. [67]	The authors reveal a significantly lower mean DTF in patients requiring reintubation versus patients who did not (12% vs. 20%, *p* = 0.008)
	Samanta et al. [68]	DTF assessment of the right diaphragm during pressure support ventilation (PSV) may be employed to predict successful weaning prior to a T-piece SBT
	Blumhof et al. [69]	A DTF > 20% is a robust predictor of extubation success during pressure support ventilation tests with 5/5 and 10/5 cmH_2_O with sensitivity and specificity values of 84.6% and 79.0%, 88.9% and 75.0%, respectively
	DiNino et al. [70]	The authors find that a DTF threshold > 30% at end inspiration has a positive predictive value of 91% and a negative predictive value of 63% for extubation success (sensitivity 88% and specificity 91%, respectively), with similar results during both SBT and pressure support tests
Diaphragm excursion (DE) and diaphragm thickening fraction (DTF)	Farghaly et al. [71]	The authors found that cut-off values of DE ≥ 10.5 mm, Te-insp ≥ 21 mm, and DTF > 34.2% during SBT with pressure support were associated with successful weaning; combining a DE ≥ 10.5 mm with a Te-insp. ≥ 21 mm decreased the sensitivity to 64.9%, but increased specificity up to 100% for successful weaning
	Zambon et al. [72]	The authors describe both DE and DTF to be useful weaning predictors, but only DTF is shown to be an accurate index of diaphragmatic contractility workload
	Llamas-Alvarez et al. [73]	This systematic review and metanalysis concludes that diaphragm ultrasound measures (DTF > DE) can help predict weaning outcome. It also suggests that the accuracy of DTF and DE may be overestimated in the current literature depending on the patient subpopulation studied
	Vivier et al. [53]	The authors conducted a multicenter prospective study involving 191 patients where DTF and DE data were collected during T-piece SBTs. They found that diaphragmatic dysfunction as assessed by ultrasound (described as DE < 10 mm and DTF < 30%) was not associated with extubation failure

*DUS* diaphragm ultrasound, *DE* diaphragm excursion, *DTF* diaphragm thickening fraction, *SBT* spontaneous breathing trial, *Te-insp.* end-inspiratory thickness

Although bilateral anterior magnetic stimulation of the phrenic nerves is considered the gold standard for the assessment of diaphragm function [48], it requires high expertise and expensive machines. By contrast, ultrasonography is a widely available non-invasive tool that provides both structural and functional information about the muscle. Regarding diaphragm evaluation by ultrasonography and the therapeutic and prognostic implications of this measure, more studies are needed to evaluate the accuracy of ultrasound measures of the diaphragm and their therapeutic and prognostic implications and applicability in mechanical ventilation (i.e., diaphragm-protective ventilation and weaning from IMV). Further research is also required to standardize the measurement techniques and teaching programs [74].

## Conclusions

In the first section of this review, two of the five factors involved in successful weaning from mechanical ventilation were addressed. The remaining components will be discussed in the second section of this review entitled: “Mechanical ventilation weaning issues can be counted on the fingers of just one hand: part 2”.

## Data Availability

Not applicable.

## Abbreviations

IMV: Invasive mechanical ventilation
ICU: Intensive care unit
SBT: Spontaneous breathing trial
PEEP: Positive end-expiratory pressure
GCS: Glasgow Coma Scale
RSBI: Rapid shallow breathing index
PAC: Pulmonary artery catheter
LVEF: Left ventricular ejection fraction
LVEDP: Left ventricular end-diastolic pressure
BNP: Brain natriuretic peptide
DUS: Diaphragm ultrasound
Pdi: Trans-diaphragmatic pressure
DE: Diaphragmatic excursion
DTF: Diaphragm thickening fraction
Te-insp: End-inspiration thickness
Te-exp: End expiration thickness
EAdi: Electrical activity of the diaphragm
NAVA: Neurally adjusted ventilator assist

## References

[1] Béduneau G, Pham T, Schortgen F, Piquilloud L, Zogheib E, Jonas M, Grelon F, Runge I, Terzi N, Grangé S, Barberet G, Guitard PG, Frat JP, Constan A, Chretien JM, Mancebo J, Mercat A, Richard JM, Brochard L, WIND (Weaning according to a New Definition) Study Group and the REVA (Réseau Européen de Recherche en Ventilation Artificielle) Network (2017). Epidemiology of weaning outcome according to a new definition. The WIND study. Am J Respir Crit Care Med.

[2] Thille AW, Cortés-Puch I, Esteban A (2013). Weaning from the ventilator and extubation in ICU. Curr Opin Crit Care.

[3] Boles JM, Bion J, Connors A, Herridge M, Marsh B, Melot C, Pearl R, Silverman H, Stanchina M, Vieillard-Baron A, Welte T (2007). Weaning from mechanical ventilation. Eur Respir J.

[4] Peñuelas O, Frutos-Vivar F, Fernández C, Ventila Group (2011). Characteristics and outcomes of ventilated patients according to time to liberation from mechanical ventilation. Am J Respir Crit Care Med.

[5] Levine S, Nguyen T, Taylor N, Friscia ME, Budak MT, Rothenberg P, Zhu J, Sachdeva R, Sonnad S, Kaiser LR, Rubinstein NA, Powers SKSJ (2008). Rapid disuse atrophy of diaphragm fibers in mechanically ventilated humans. N Engl J Med.

[6] Caroleo S, Agnello F, Abdallah K, Santangelo E, Amantea B (2007). Weaning from mechanical ventilation: an open issue. Minerva Anestesiol.

[7] Bruni A, Garofalo E, Pelaia C, Messina A, Cammarota G, Murabito P, Corrado S, Vetrugno L, Longhini F, Navalesi P (2019). Patient-ventilator asynchrony in adult critically ill patients. Minerva Anestesiol.

[8] Lemaire F, Teboul JL, Cinotti L, Giotto G, Abrouk F, Steg G, Macquin-Mavier I, Zapol WM (1988). Acute left ventricular dysfunction during unsuccessful weaning from mechanical ventilation. Anesthesiology.

[9] Ouellette DR, Patel S, Girard TD, Morris PE, Schmidt GA, Truwit JD, Alhazzani W, Burns SM, Epstein SK, Esteban A, Fan E, Ferrer M, Fraser GL, Gong MN, Hough CL, Mehta S, Nanchal R, Pawlik AJ, Schweickert WD, Sessler CN, Strøm T, Kress JP (2017). Liberation from mechanical ventilation in critically ill adults: an Official American College of Chest Physicians/American Thoracic Society clinical practice guideline: inspiratory pressure augmentation during spontaneous breathing trials, protocols minimizing sedation, and noninvasive ventilation immediately after extubation. Chest.

[10] Esteban A, Alia I, Gordo F, Fernandex R, Solsona J, Vallverdu I, Macías S, Allegue JM, Blanco J, Carriedo D, León M, de la Cal MA, Taboada F, Gonzalez de Velasco J, Palazón E, Carrizosa F, Tomás R, Suarez J, Goldwasser RS (1997). Extubation outcome after spontaneous breathing trials with t-tube or pressure support ventilation. Am J Respir Crit Care Med.

[11] Esteban A, Alía I, Tobin MJ, Gil A, Gordo F, Vallverdú I, Blanch L, Bonet A, Vázquez A, de Pablo R, Torres A, de La Cal MA, Macías S (1999). Effect of spontaneous breathing trial duration on outcome of attempts to discontinue mechanical ventilation. Am J Respir Crit Care Med.

[12] Frutos-Vivar F, Ferguson ND, Esteban A, Epstein SK, Arabi Y, Apezteguía C, González M, Hill NS, Nava S, D’Empaire G, Anzueto A (2006). Risk factors for extubation failure in patients following a successful spontaneous breathing trial. Chest.

[13] Ouanes-Besbes L, Dachraoui F, Ouanes I, Bouneb R, Jalloul F, Dlala M, Najjar MF, Abroug F (2012). NT-proBNP levels at spontaneous breathing trial help in the prediction of post-extubation respiratory distress. Intensive Care Med.

[14] Meade M, Guyatt G, Cook D, Griffith L, Sinuff T, Kergl C, Mancebo J, Esteban A, Epstein S (2001). Predicting success in weaning from mechanical ventilation. Chest.

[15] Conti G, Montini L, Pennisi MA, Cavaliere F, Arcangeli A, Bocci MG, Proietti R, Antonelli M (2004). A prospective, blinded evaluation of indexes proposed to predict weaning from mechanical ventilation. Intensive Care Med.

[16] Thille AW, Richard JC, Brochard L (2013). The decision to extubate in the intensive care unit. Am J Respir Crit Care Med.

[17] Silva S, Ait Aissa D, Cocquet P, Hoarau L, Ruiz J, Ferre F, Rousset D, Mora M, Mari A, Fourcade O, Riu B, Jaber S, Bataille B (2017). Combined thoracic ultrasound assessment during a successful weaning trial predicts postextubation distress. Anesthesiology.

[18] Aybar Türkoğlu M, Topeli Iskit A (2008). Ventilator-associated pneumonia caused by high risk microorganisms: a matched case-control study. Tuberk Toraks.

[19] Soler Morejón Cde D, Tamargo Barbeito TO (2012). Effect of mechanical ventilation on intra-abdominal pressure in critically ill patients without other risk factors for abdominal hypertension: an observational multicenter epidemiological study. Ann Intensive Care.

[20] Namen AM, Ely EW, Tatter SB, Case LD, Lucia MA, Smith A, Landry S, Wilson JA, Glazier SS, Branch CL, Kelly DL, Bowton DL, Haponik EF (2001). Predictors of successful extubation in neurosurgical patients. Am J Respir Crit Care Med.

[21] Peñuelas Ó, Thille AW, Esteban A (2015). Discontinuation of ventilatory support: new solutions to old dilemmas. Curr Opin Crit Care.

[22] Burns KEA, Soliman I, Adhikari NKJ, Zwein A, Wong JTY, Gomez-Builes C, Pellegrini JA, Chen L, Rittayamai N, Sklar M, Brochard LJ, Friedrich JO (2017). Trials directly comparing alternative spontaneous breathing trial techniques: a systematic review and meta-analysis. Crit Care.

[23] MacIntyre NR (2013). The ventilator discontinuation process: an expanding evidence base. Respir Care.

[24] Malbrain MLNG, Van Regenmortel N, Saugel B, De Tavernier B, Van Gaal PJ, Joannes-Boyau O, Teboul JL, Rice TW, Mythen M, Monnet X (2018). Principles of fluid management and stewardship in septic shock: it is time to consider the four D’s and the four phases of fluid therapy. Ann Intensive Care.

[25] Sakr Y, Rubatto Birri PN, Kotfis K, Nanchal R, Shah B, Kluge S, Schroeder ME, Marshall JC, Vincent JL, Intensive Care Over Nations Investigators (2017). Higher fluid balance increases the risk of death from sepsis: results from a large international audit. Crit Care Med.

[26] Boyd JH, Forbes J, Nakada TA, Walley KR, Russell JA (2011). Fluid resuscitation in septic shock: a positive fluid balance and elevated central venous pressure are associated with increased mortality. Crit Care Med.

[27] Franchi F, Vetrugno L, Scolletta S (2017). Echocardiography to guide fluid therapy in critically ill patients: check the heart and take a quick look at the lungs. J Thorac Dis.

[28] Vignon P, Repessé X, Viellard-Baron A, Maury E (2016). Critical care ultrasonography in acute respiratory failure. Crit Care.

[29] Epstein SK (1995). Etiology of extubation failure and the predictive value of the rapid shallow breathing index. Am J Resp Crit Care Med.

[30] Pandey A, Khera R, Kumar N, Golwala H, Girotra S, Fonarow GC (2016). Use of pulmonary arterial catheterization in US patients with failure, 2001–2012. JAMA Intern Med.

[31] Della Rocca G, Vetrugno L, Tripi G, Deana C, Barbariol F, Pompei L (2014). Liberal or restricted fluid administration: are we ready for a proposal of a restricted intraoperative approach?. BMC Anesthesiol.

[32] Lamia B, Maizel J, Ochagavia A, Chemla D, Osman D, Richard C, Teboul JL (2009). Echocardiographic diagnosis of pulmonary artery occlusion pressure elevation during weaning from mechanical ventilation. Crit Care Med.

[33] Ommen SR, Nishimura RA, Appleton CP, Miller FA, Oh JK, Redfield MM, Tajik AJ (2000). Clinical utility of Doppler echocardiography and tissue doppler imaging in the estimation of left ventricular filling pressures: a comparative simultaneous Doppler-catheterization study. Circulation.

[34] Moschietto S, Doyen D, Grech L, Dellamonica J, Hyvernat H, Bernardin G (2012). Transthoracic echocardiography with Doppler tissue imaging predicts weaning failure from mechanical ventilation: evolution of the left ventricle relaxation rate during a spontaneous breathing trial is the key factor in weaning outcome. Crit Care.

[35] Nagueh S, Smiseth O, Appleton C, Byrd B, Dokainish H, Edvardsen T, Flachskampf FA, Gillebert TC, Klein AL, Lancellotti P, Marino P, Oh JK, Popescu BA, Waggoner AD (2016). Recommendations for the evaluation of left ventricular diastolic function by echocardiography: an update from the American Society of Echocardiography and the European Association of Cardiovascular Imaging. J Am Soc Echocardiogr.

[36] Ferré A, Guillot M, Lichtenstein D, Mezière G, Richard C, Teboul JL, Monnet X (2019). Lung ultrasound allows the diagnosis of weaning-induced pulmonary oedema. Intensive Care Med.

[37] Mekontso Dessap A, Roche-Campo F, Kouatchet A, Tomicic V, Beduneau G, Sonneville R, Cabello B, Jaber S, Azoulay E, Castanares-Zapatero D, Devaquet J, Lellouche F, Katsahian S, Brochard L (2012). Natriuretic peptide-driven fluid management during ventilator weaning: a randomized controlled trial. Am J Respir Crit Care Med.

[38] Robertson CH, Foster GM, Johnson RL (1977). The relationship of respiratory failure to the oxygen consumption of, lactate production by, and distribution of blood flow among respiratory muscles during increasing inspiratory resistance. J Clin Investig.

[39] Rochester DF, Pradel-Guena H (1973). Measurement of diaphragmatic blood flow in dogs from Xenon-133 clearance. J Appl Physiol.

[40] Monod H, Scherrer J (1965). The work capacity of a synergic muscular group. Ergonomics.

[41] Aubier M, Viires N, Syllie G, Mozes R, Roussos CS (1982). Respiratory muscle contribution to lactic acidosis in low cardiac output. Am Rev Respir Dis.

[42] Macklem PT, Roussos CS (1977). Respiratory muscle fatigue: a cause of respiratory failure?. Clin Sci Mol Med.

[43] Kim WY, Suh HJ, Hong SB, Koh Y, Lim CM (2011). Diaphragm dysfunction assessed by ultrasonography: influence on weaning from mechanical ventilation. Crit Care Med.

[44] Powers SK, Kavazis AN, Levine S (2009). Prolonged mechanical ventilation alters diaphragmatic structure and function. Crit Care Med.

[45] Vetrugno L, Guadagnin GM, Barbariol F, Langiano N, Zangrillo A, Bove T (2019). Ultrasound imaging for diaphragm dysfunction: a narrative literature review. J Cardiothorac Vasc Anesth.

[46] Dres M, Dubé BP, Mayaux J, Delemazure J, Reuter D, Brochard L, Similowski T, Demoule A (2017). Coexistence and impact of limb muscle and diaphragm weakness at time of liberation from mechanical ventilation in medical intensive care unit patients. Am J Respir Crit Care Med.

[47] Lerolle N, Guérot E, Dimassi S, Zegdi R, Faisy C, Fagon JY, Diehl JL (2009). Ultrasonographic diagnostic criterion for severe diaphragmatic dysfunction after cardiac surgery. Chest.

[48] Watson AC, Hughes PD, Louise Harris M, Hart N, Ware RJ, Wendon J, Green M, Moxham J (2001). Measurement of twitch transdiaphragmatic, esophageal, and endotracheal tube pressure with bilateral anterolateral magnetic phrenic nerve stimulation in patients in the intensive care unit. Crit Care Med.

[49] Dubé BP, Dres M (2016). Diaphragm dysfunction: diagnostic approaches and management strategies. J Clin Med.

[50] Bignami E, Guarnieri M, Saglietti F, Ramelli A, Vetrugno L (2018). Diaphragmatic dysfunction following cardiac surgery: is there a role for pulmonary ultrasound?. J Cardiothorac Vasc Anesth.

[51] Barbariol F, Vetrugno L, Pompei L, De Flaviis A, Rocca GD (2015). Point-of-care ultrasound of the diaphragm in a liver transplant patient with acute respiratory failure. Crit Ultrasound J.

[52] Cammarota G, Sguazzotti I, Zanoni M, Messina A, Colombo D, Vignazia GL, Vetrugno L, Garofalo E, Bruni A, Navalesi P, Avanzi GC, Della Corte F, Volpicelli G, Vaschetto R (2019). Diaphragmatic ultrasound assessment in subjects with acute hypercapnic respiratory failure admitted to the emergency department. Respir Care.

[53] Vivier E, Muller M, Putegnat JB, Steyer J, Barrau S, Boissier F, Bourdin G, Mekontso-Dessap A, Levrat A, Pommier C, Thille AW (2019). Inability of diaphragm ultrasound to predict extubation failure: a multicenter study. Chest.

[54] Boussuges A, Gole Y, Blanc P (2009). Diaphragmatic motion studied by M-mode ultrasonography: methods, reproducibility, and normal values. Chest.

[55] Summerhill EM, El-Sameed YA, Glidden TJ, McCool FD (2008). Monitoring recovery from diaphragm paralysis with ultrasound. Chest.

[56] Wait JL, Nahormek PA, Yost WT, Rochester DP (1989). Diaphragmatic thickness-lung volume relationship in vivo. J Appl Physiol.

[57] Cohen E, Mier A, Heywood P, Murphy K, Boultbee J, Guz A (1994). Excursion-volume relation of the right hemidiaphragm measured by ultrasonography and respiratory airflow measurements. Thorax.

[58] Umbrello M, Formenti P, Longhi D, Galimberti A, Piva I, Pezzi A, Mistraletti G, Marini JJ, Iapichino G (2015). Diaphragm ultrasound as indicator of respiratory effort in critically ill patients undergoing assisted mechanical ventilation: a pilot clinical study. Crit Care.

[59] Goligher EC, Fan E, Herridge MS, Murray A, Vorona S, Brace D, Rittayamai N, Lanys A, Tomlinson G, Singh JM, Bolz SS, Rubenfeld GD, Kavanagh BP, Brochard LJ, Ferguson ND (2015). Evolution of diaphragm thickness during mechanical ventilation: impact of inspiratory effort. Am J Respir Crit Care Med.

[60] Dubé BP, Dres M, Mayaux J, Demiri S, Similowski T, Demoule A (2017). Ultrasound evaluation of diaphragm function in mechanically ventilated patients: comparison to phrenic stimulation and prognostic implications. Thorax.

[61] Jiang JR, Tsai TH, Jerng JS, Yu CJ, Wu HD, Yang PC (2004). Ultrasonographic evaluation of liver/spleen movements and extubation outcome. Chest.

[62] Spadaro S, Grasso S, Mauri T, Dalla Corte F, Alvisi V, Ragazzi R, Cricca V, Biondi G, Di Mussi R, Marangoni E, Volta CA (2016). Can diaphragmatic ultrasonography performed during the T-tube trial predict weaning failure? The role of diaphragmatic rapid shallow breathing index. Crit Care.

[63] Luo L, Li Y, Chen X, Sun B, Li W, Gu W, Wang S, Zhao S, Lv Y, Chen M, Xia J, Sui F, Mei X, Shi H, Tong Z (2017). Different effects of cardiac and diaphragm function assessed by ultrasound on extubation outcomes in difficult-to-wean patients: a cohort study. BMC Pulm Med.

[64] Garrido-Aguirre E, Ñamendys-Silva SA, del Moral OR, Cortés-Soto CA, Romero-González JP (2019). Diaphragmatic ultrasonography, a novel approach in critical care. A proposal for a new weaning index. Ultrasound Q.

[65] Palkar A, Narasimhan M, Greenberg H, Singh K, Koenig S, Mayo P, Gottesman E (2018). Diaphragm excursion-time index. A new parameter using ultrasonography to predict extubation outcome. Chest.

[66] Carrie C, Gisbert-Mora C, Bonnardel E, Gauche B, Biais M, Vargas F, Hilbert G (2017). Ultrasonographic diaphragmatic excursion is inaccurate and not better than the MRC score for predicting weaning-failure in mechanically ventilated patients. Anaesth Crit Care Pain Med.

[67] Jung B, Moury PH, Mahul M, de Jong A, Galia F, Prades A, Albaladejo P, Chanques G, Molinari N, Jaber S (2016). Diaphragmatic dysfunction in patients with ICU-acquired weakness and its impact on extubation failure. Intensive Care Med.

[68] Samanta S, Singh RK, Baronia AK, Poddar B, Azim A, Gurjar M (2017). Diaphragm thickening fraction to predict weaning—a prospective exploratory study. J Intensive Care.

[69] Blumhof S, Wheeler D, Thomas K, McCool FD, Mora J (2016). Change in diaphragmatic thickness during the respiratory cycle predicts extubation success at various levels of pressure support ventilation. Lung.

[70] DiNino E, Gartman EJ, Sethi JM, McCool FD (2014). Diaphragm ultrasound as a predictor of successful extubation from mechanical ventilation. Thorax.

[71] Farghaly S, Hasan AA (2017). Diaphragm ultrasound as a new method to predict extubation outcome in mechanically ventilated patients. Aust Crit Care.

[72] Zambon M, Greco M, Bocchino S, Cabrini L, Beccaria PF, Zangrillo A (2017). Assessment of diaphragmatic dysfunction in the critically ill patient with ultrasound: a systematic review. Intensive Care Med.

[73] Llamas-Álvarez AM, Tenza-Lozano EM, Latour-Pérez J (2017). Diaphragm and lung ultrasound to predict weaning outcome: systematic review and meta-analysis. Chest.

[74] Garofalo E, Bruni A, Pelaia C, Landoni G, Zangrillo A, Antonelli M, Conti G, Biasucci DG, Mercurio G, Cortegiani A, Giarratano A, Vetrugno L, Bove T, Forfori F, Corradi F, Vaschetto R, Cammarota G, Ma A, Murabito P, Bellini V, Zambon M, Longhini F, Navalesi P, Bignami E (2019). Comparisons of two diaphragm ultrasound-teaching programs: a multicenter randomized controlled educational study. Ultrasound J.

